# A nested mixture model for genomic prediction using whole-genome SNP genotypes

**DOI:** 10.1371/journal.pone.0194683

**Published:** 2018-03-21

**Authors:** Jian Zeng, Dorian Garrick, Jack Dekkers, Rohan Fernando

**Affiliations:** 1 Institute for Molecular Bioscience, The University of Queensland, Brisbane, Queensland, Australia; 2 School of Agriculture, Massey University, Palmerston North, New Zealand; 3 Department of Animal Science, Iowa State University, Ames, Iowa, United States of America; China Agricultural University, CHINA

## Abstract

Genomic prediction exploits single nucleotide polymorphisms (SNPs) across the whole genome for predicting genetic merit of selection candidates. In most models for genomic prediction, e.g. BayesA, B, C, R and GBLUP, independence of SNP effects is assumed. However, SNP effects are expected to be locally dependent given the presence of a nearby QTL because SNPs surrounding the QTL do not segregate independently. A consequence of ignoring this dependence is that SNPs with small effects may be overly shrunk, e.g. effects from markers with high minor allele frequencies (MAF) that flank QTL with low MAF. A nested mixture model (BayesN) is developed to account for the dependence of effects of SNPs that are closely linked, where the effects of SNPs in every non-overlapping genomic window *a priori* follow a point mass at zero for all SNPs or a mixture of some SNPs with nonzero effects and others with zero effects. It can be regarded as a parsimonious alternative to the existing antedependence model, antiBayesB, which allow a nonstationary dependence of SNP effects. Illumina 777K BovineHD genotypes from 948 Angus cattle were used to simulate 5,000 offspring, with 4,000 used for training and 1,000 for validation. Scenarios with 300 common (MAF > 0.05) or rare (MAF < 0.05) QTL randomly selected from segregating SNPs were replicated 8 times. SNPs corresponding to QTL were masked from a 600k panel comprising SNPs with MAF > 0.05 or a 50k evenly spaced subset of these. Compared with BayesB and a modified antiBayesB, BayesN improved the accuracy of prediction up to 2.0% with 50k SNPs and up to 7.0% with 600k SNPs, most improvements occurring in the rare QTL scenario. Computing time was reduced up to 60% with 50k SNPs and up to 75% with 600k SNPs. BayesN is an accurate and computationally efficient method for genomic prediction with whole-genome SNPs, especially for traits with rare QTL.

## Introduction

Genomic prediction exploits single-nucleotide polymorphisms (SNPs) across the whole genome for predicting genetic merit of selection candidates. It has been successfully applied in animal breeding using panels with about 50k SNPs (medium-density, MD) [1–3]. High-density (HD) SNP panels, such as those with more than 500k SNPs, are expected to include a large number of SNPs that are physically proximal to every causal variant or quantitative trait locus (QTL). An HD panel must contain a subset of informative markers that provide more accurate prediction than an equivalent number of markers from an MD panel. The challenge is to develop a prediction equation that derives most of its predictive ability from that informative subset. The advantage of using HD rather than MD SNP panels has been small in practice [4–6], demonstrating either insufficient data or inadequacies of currently applied methods.

One possible explanation for the failure to improve predictive ability using an HD rather than MD panel is that the QTL underlying the trait may have low minor allele frequency (MAF), whereas the markers are typically chosen to avoid low MAF [7]. In the absence of balancing selection, ancient mutations will have low MAF, as will any recent mutation. High linkage disequilibrium (LD) cannot exist between a SNP and QTL that have widely varying allele frequencies [8]. Such a SNP alone cannot capture the effect of QTL with rare alleles.

Compared to QTL with high MAF, QTL with low MAF will need more SNPs to jointly capture the QTL effect. Informative priors can be used to allow multiple SNPs that are linked to the QTL to jointly capture its effect. As shown in S1 Appendix, the cosegregation of QTL and linked SNPs will result in these SNP effects being dependent. Modeling such dependence of SNP effects in the prior will improve the ability the SNPs close to a QTL to jointly capture its effect. Note that in most models used for genomic prediction [9–13], SNP effects are assumed to be independently and identically distributed. For instance, the BayesB model [9] assumes a prior distribution that is an *i*.*i*.*d*. mixture of a point mass at zero and a *t*-distribution for each SNP effect to accommodate situations where only a fraction of SNPs have nonzero effects. Priors that model the dependence of SNP effects allow SNPs surrounding a QTL to jointly capture its effect better than priors that assume independence of SNP effects.

Furthermore, the dependence between SNP effects is expected to increase with panel density, in that the multi-locus LD with the QTL increases, although the pairwise LD between single SNPs and QTL may be low for QTL with rare alleles. In mixture models that ignore the dependence of SNP effects, the prior probability that a SNP has a nonzero effect decreases as panel density increases, since the number of QTL is a characteristic of the trait and population but not of the panel density. As a consequence, the effects of SNPs with small effects will be shrunk toward zero, as the prior probability that a SNP has nonzero effect decreases. But in models that account for the dependence of SNP effects, the effects of SNPs that are linked to a QTL are expected to be shrunk less because modeling the dependence of SNP effects will facilitate these SNPs to be included in the model jointly.

Gianola *et al*. [14] and Yang and Tempelman [13] have proposed models to account for the dependence of SNP effects along the chromosome. They were motivated by the evidence of coexpression of genes on the same chromosome [14, 15]. Coexpression of QTL will increase dependence of their effects, and thus, the effects of QTL and those closely linked SNPs are expected to be correlated [14]. However, as discussed above, SNP effects can be correlated even without coexpression due to cosegregation of SNP and QTL alleles. Gianola *et al*. [14] proposed several approaches to model the covariance between marker effects, but all of them have a stationary structure [13]. The model of Yang and Tempelman [13] is a first-order antedependence model that allows for a nonstationary covariance structure. In their model, besides the first SNP on a chromosome, each following SNP has an independent effect in addition to a regression on the effect of its anterior neighbor. Thus, the number of effects in the model is twice the number of SNPs on a chromosome minus one. This doubles the number of effects to be estimated. The BayesB model with antedependence covariance structure was referred to as anteBayesB [13].

In this study, a nested mixture model (BayesN) is developed to account for the dependence of effects for SNPs that are closely linked. The effects of SNPs within an arbitrary non-overlapping chromosomal segment, e.g. 1 or 0.2 megabase (Mb), are collectively considered as a window. The prior for the collective SNP effects in window *i* follows a point mass at zero for all SNPs with known or unknown probability Π or a mixture of some SNPs with nonzero effects and others with zero effects with probability (1 − Π). The dependence of SNP effects is modeled through such a window hierarchy. Furthermore, the Markov chain Monte Carlo (MCMC) sampling is expected to be more efficient with BayesN because with probability Π all SNPs in the window are sampled to have zero effects, instead of sampling each SNP effect from a mixture distribution as in BayesB. This benefit will increase with SNP density and be greatest for genomic prediction using next-generation sequence density.

The objective of this paper is to introduce the nested model and compare its predictive and computational performance with that of anteBayesB and BayesB. Training and validation populations were simulated offspring derived from 948 Aberdeen Angus cattle that had been genotyped using the Illumina 777k BovineHD BeadChip. Two scenarios corresponding to trait variation being determined by QTL with common or rare MAF were investigated for each of two different SNP densities, corresponding to 50k and 600k panels.

## Methods

The general model for genomic prediction is a mixed linear model [9, 16]:

$$\begin{array}{c} y=X\beta +Zu+e, \end{array}$$ (1)

where **y** is a vector of *n* phenotypes, **X** is an incidence matrix for the fixed effects, ***β*** is a vector of fixed effects with a flat prior, **Z** is an *n* × *m* matrix of SNP genotype scores ∈{0, 1, 2}, **u** is a vector of random effects of *m* SNPs with mixture priors, and **e** is a vector of residuals with each element $∼N(0,\sigma_{e}^{2})$ with prior distribution for $\sigma_{e}^{2}$ is $\nu_{e}S_{e}^{2}\chi_{\nu_{e}}^{-2}$, where scale factor $S_{e}^{2}=\frac{V_{E}\left(\nu_{e}-2\right)}{\nu_{e}}$ for a given residual variance *V*_*E*_ and *ν*_*e*_ is the degrees of freedom associated with the prior. In this study, the only fixed effect was the population mean. Complete specification of the model requires prior specification of the SNP effects, and there are a number of commonly used alternative priors [9–13]. First, the prior specification for BayesB, which assumes independence of SNP effects, is briefly introduced, and then the prior specifications for BayesN and anteBayesB, which account for the dependence of SNP effects, are described.

### BayesB

It is assumed that each SNP effect in Eq (1) is independently and identically distributed as:

$$\begin{array}{c} u_{j}\overset{i.i.d.}{∼}\{\begin{array}{cc} \begin{array}{c} 0,\\ t_{\nu_{\alpha }}\left(0,S_{\alpha }^{2}\right), \end{array} & \begin{array}{cc} \text{with}\;\text{probability} & \pi ,\\ \text{with}\;\text{probability} & 1-\pi , \end{array} \end{array} \end{array}$$

where the degrees of freedom *ν*_*α*_, the scale factor $S_{\alpha }^{2}$ and the probability *π* are assumed known hyperparameters.

Following [16], for computational convenience, *u*_*j*_ can be written as the product of a t-variable and a Bernoulli indicator variable:

$$\begin{array}{c} u_{j}=\alpha_{j}\delta_{j}. \end{array}$$ (2)

The prior for *α*_*j*_ is a normal distribution conditional on a locus-specific variance $\sigma_{j}^{2}$, which has a scaled inverse Chi-square distribution [9]:

$$\begin{array}{c} \alpha_{j}|\sigma_{j}^{2}∼N\left(0,\sigma_{j}^{2}\right), \end{array}$$

$$\begin{array}{c} \sigma_{j}^{2}∼\nu_{\alpha }S_{\alpha }^{2}\chi_{\nu_{\alpha }}^{-2}. \end{array}$$

The resulting marginal distribution for *α*_*j*_ is a univariate-t [17]:

$$\begin{array}{c} \alpha_{j}∼t_{\nu_{\alpha }}\left(0,S_{\alpha }^{2}\right). \end{array}$$

A fat-tailed t-distribution with *ν*_*α*_ = 4 was chosen to accommodate some SNPs with large effects. The value of $S_{\alpha }^{2}$ is calculated as

$$\begin{array}{ccc} S_{\alpha }^{2} & = & \frac{V_{A}}{2\bar{pq}\left(1-\pi \right)m}\frac{\nu_{\alpha }-2}{\nu_{\alpha }}, \end{array}$$ (3)

where *V*_*A*_ is the additive genetic variance and $\bar{pq}$ is the mean product of alternate allele frequencies for all SNPs [16]. Simplification of this prior for *α*_*j*_ that specifies a common variance for all SNPs, $\alpha_{j}|\sigma_{\alpha }^{2}∼N(0,\sigma_{\alpha }^{2})$, is known as BayesC [10].

The prior for *δ*_*j*_ is,

$$\begin{array}{c} \delta_{j}=\{\begin{array}{cc} \begin{array}{c} 0\\ 1 \end{array} & \begin{array}{cc} \text{with}\;\text{probability} & \pi ,\\ \text{with}\;\text{probability} & 1-\pi . \end{array} \end{array} \end{array}$$

Usually, for *m* ≫ *n*, a value close to one is given to *π* to account for the fact that the majority of SNPs are not expected to be associated with the trait. To better demonstrate the meaning of *π*, we calculated it based on the number s of QTL obtained from the simulation, assuming that on average each QTL is associated with *k* = 2 or 10 SNPs,

$$\begin{array}{c} \pi =\frac{m-s\cdot k}{m}. \end{array}$$ (4)

We will see later that this expression for *π* is also useful to compare methods at the same level of *π*. In practice, there may not be sufficient information on the values of *s* and *k*. However, *π* can be considered as unknown with a uniform prior between zero and one. But, we have observed that BayesB with this prior has a mixing problem when sample size is small. Thus, Habier *et al*. [11] used BayesC with *π* was treated as unknown, and this approach is called BayesC*π*. Thus, the posterior mean of *π* from BayesC*π* is often used as the value of *π* in BayesB [18–20].

Substituting *u*_*j*_ by *α*_*j*_ *δ*_*j*_, Eq (1) can be written as

$$\begin{array}{c} y=X\beta +\sum_{j=1}^{m}Z_{j}\alpha_{j}\delta_{j}+e. \end{array}$$ (5)

It is straightforward to sample *α*_*j*_ and *δ*_*j*_ from their full conditional distributions with this model as both have closed forms [16].

### BayesN

According to the map positions of SNPs, SNP effects are nested within non-overlapping windows. As described below, hierarchical mixtures at the window and SNP levels are used to account for the dependence of SNP effects within a window but independence between windows. Let Δ_*i*_ be a Bernoulli indicator specifying whether window *i* is in or out of the model. As in Eq (2), the effect *u*_*ij*_ for SNP *j* in window *i* can then be parameterized as the product of three variables:

$$\begin{array}{c} u_{ij}=\alpha_{ij}\delta_{ij}\Delta_{i}. \end{array}$$ (6)

The prior for *α*_*ij*_ is a normal conditional on a window-specific common variance $\sigma_{i}^{2}$ that has a scaled inverse Chi-square distribution:

$$\begin{array}{c} \alpha_{ij}|\sigma_{i}^{2}∼N\left(0,\sigma_{i}^{2}\right), \end{array}$$

$$\begin{array}{c} \sigma_{i}^{2}∼\nu_{\alpha }S_{\alpha }^{2}\chi_{\nu_{\alpha }}^{-2}, \end{array}$$

which results in a multivariate-t distribution [17] for ***α***_*i*_:

$$\begin{array}{c} \alpha_{i}∼t_{\nu_{\alpha }}\left(0,IS_{\alpha }^{2}\right). \end{array}$$

With this prior, the effects are uncorrelated but dependent. BayesN is similar to BayesB in that the conditional prior for the window effect has a window-specific variance, just as the conditional prior for the SNP effect in BayesB has a locus-specific variance. On the other hand, BayesN is similar to BayesC in that the conditional prior for effects within a window have a common variance just as the conditional prior for SNP effects in BayesC have a common variance.

The prior for the locus effect indicator in Eq (6) is

$$\begin{array}{c} \delta_{ij}=\{\begin{array}{cc} \begin{array}{c} 0\\ 1 \end{array} & \begin{array}{cc} \text{with}\;\text{probability} & \pi_{i},\\ \text{with}\;\text{probability} & 1-\pi_{i}, \end{array} \end{array} \end{array}$$

where *π*_*i*_ is the window-specific probability calculated as

$$\begin{array}{c} \pi_{i}=\frac{m_{i}-k}{m_{i}}, \end{array}$$

where *m*_*i*_ is the number of SNPs in window *i*, and *k* is the number of SNPs to be fitted per window. Assuming a window contains at most one QTL, *k* is the average number of SNPs associated with a QTL, as defined in Eq (4). The window-specific probability accounts for the variability of SNP density across windows. It allows the probability of inclusion for the SNP effect to be invariant to the SNP density outside the window.

The prior for the window effect indicator in Eq (6) is

$$\begin{array}{c} \Delta_{i}=\{\begin{array}{cc} \begin{array}{c} 0\\ 1 \end{array} & \begin{array}{cc} \text{with}\;\text{probability} & \Pi ,\\ \text{with}\;\text{probability} & 1-\Pi , \end{array} \end{array} \end{array}$$

where Π is the proportion of windows that contain no QTL. Let *w* denote the total number of windows in the genome. Again with the assumption of at most one QTL per window,

$$\begin{array}{c} \Pi =\frac{w-s}{w}, \end{array}$$

where *s* is as defined in Eq (4). The window effect indicator introduces another source of dependence for the SNP effects by forcing SNPs in a window to be fitted or dropped out of the model jointly.

It can be shown that with these specifications for *π*_*i*_ and Π, when the number of SNPs in the window *m*_*i*_ is constant across all windows, the prior proportion of nonzero effects will be equivalent to that specified in BayesB given the same value of *k*:

$$\begin{array}{cc} \left(1-\Pi )(1-\pi_{i}\right) & =\frac{s\cdot k}{w\cdot m_{i}}\\ & =\frac{s\cdot k}{m}\\ & =1-\pi . \end{array}$$

As in BayesC*π*, Π can be considered as unknown with an uniform prior. Because mixing was an issue when BayesB was used with an unknown *π*, we conjectured that BayesN with window-specific variances may run into a similar mixing problem. Thus, results from BayesN with window-specific variance $\sigma_{i}^{2}$ is compared to those from BayesN with a common variance $\sigma_{\alpha }^{2}$ for all SNPs in the genome, which is referred to BayesNC, when Π is treated as unknown.

For BayesN, Eq (1) can be written as

$$\begin{array}{c} y=X\beta +\sum_{i=1}^{w}\sum_{j=1}^{m_{i}}Z_{ij}\alpha_{ij}\delta_{ij}\Delta_{i}+e, \end{array}$$ (7)

where **Z**_*ij*_ is the vector of genotypes for SNP *j* in window *i*. The SNP will have a nonzero effect only when both *δ*_*ij*_ = 1 and Δ_*i*_ = 1.

### anteBayesB

The antedependence model described here is a modified version of anteBayesB [13]. As in BayesB, the SNP effect *u*_*j*_ can be factored into an underlying effect *α*_*j*_ and a Bernoulli indicator *δ*_*j*_. In contrast to BayesB, it is further assumed that each *α*_*j*_ can be modeled as:

$$\begin{array}{c} \alpha_{j}=\{\begin{array}{cc} \begin{array}{c} \gamma_{1}\\ t_{j}\alpha_{j-1}+\gamma_{j} \end{array} & \begin{array}{cc} \text{if} & j=1,\\ \text{if} & 2⩽j⩽m, \end{array} \end{array} \end{array}$$

or in matrix notation,

$$\begin{array}{ccc} \alpha & = & T\alpha +\gamma \\ & = & {\left(I-T\right)}^{-1}\gamma , \end{array}$$

where **T** is an *m* × *m* matrix,

$$\begin{array}{c} T=[\begin{array}{ccccc} 0 & 0 & 0 & \cdots & 0\\ t_{2} & 0 & 0 & \ddots & 0\\ 0 & t_{3} & 0 & \ddots & 0\\ \vdots & \ddots & \ddots & \ddots & 0\\ 0 & 0 & 0 & t_{m} & 0 \end{array}]. \end{array}$$

Conditional on **T**, the dependence of ***α*** is modeled by Var (***α***|**T**) = (**I** − **T**)^−1^ Var (***γ***) (**I** − **T**)^−T^ where

$$\begin{array}{c} {\left(I-T\right)}^{-1}{\left(I-T\right)}^{-\text{T}}=[\begin{array}{ccc} 1 & \cdots & \prod_{j=2}^{m}(-t_{j})\\ \vdots & \ddots & \vdots \\ \prod_{j=2}^{m}(-t_{j}) & \cdots & 1+\sum_{j=2}^{m}\prod_{k=j}^{m}t_{k}^{2} \end{array}]. \end{array}$$ (8)

The conditional covariance structure shows that the effects of SNPs on the same chromosome are mutually correlated, and since *t*_*j*_ < 1 for all *j*, the covariance between any two SNPs diminishes to zero when they are far apart in location. Since the covariance variable *t*_*j*_ is a nuisance parameter in anteBayesB, just like the variance variable $\sigma_{j}^{2}$ is a nuisance parameter in BayesB, which can be integrated out, the marginal variance-covariance structure of ***α*** is

$$\begin{array}{ll} \text{Var}(\alpha ) & =\text{E}[\text{Var}(\alpha |\text{T})]\\ & =\left[\begin{array}{ccc} 1 & & 0\\ & \ddots & \\ 0 & & 1+\sum_{j=2}^{m}\prod_{k=j}^{m}\sigma_{t}^{2} \end{array}\right]\sigma_{\gamma }^{2}. \end{array}$$

assuming *t*_*j*_ has mean zero and variance $\sigma_{t}^{2}$ and $\text{Var}(\gamma )=I\sigma_{\gamma }^{2}$. As expected (S1 Appendix), the effects of SNPs are marginally uncorrelated, but the dependence of effects still holds because the effects are not normally distributed as shown below. A downside of this model is that the variance of SNP effects increases over SNPs, although the increment becomes negligible as the product of $\sigma_{t}^{2}$ approximates to zero. This is known as a non-stationary autoregressive model for longitudinal analysis that are widely used in many fields. The covariance matrix for SNP effects **u** is sparser than that shown above for ***α***, as rows and columns are zero for SNPs with *δ*_*j*_ = 0.

We specify $\gamma_{j}∼t_{\nu_{\gamma }}\left(0,S_{\gamma }^{2}\right)$ with *ν*_*γ*_ = 4, the same as *ν*_*α*_ in BayesB, and $S_{\gamma }^{2}=S_{\alpha }^{2}$ in Eq (3). The only difference between this model and Yang and Templeman’s anteBayesB, is that they assumed a mixture distribution for *γ*_*j*_ instead of for the marginal marker effect. As a result, in their model, variable selection is less related to the number of SNPs in the model, because a SNP may still have an effect even if *γ*_*j*_ = 0, depending on the effect for the anterior SNP. The prior for *t*_*j*_ is also a t-distribution $t_{\nu_{t}}\left(0,S_{t}^{2}\right)$, with *ν*_*t*_ = 4 and $S_{t}^{2}=0.1$, such that the expected value of the variance of *t*_*j*_ is $E\left(\sigma_{t}^{2}\right)=\frac{\nu_{t}S_{t}^{2}}{\nu_{t}-2}=0.2$, following the results in their paper (S2 Fig in Yang and Templeman).

### MCMC

Gibbs sampling was used to construct a Markov chain that has a stationary distribution identical to the posterior distribution of the marker effects. The chain length was 21,000 or 101,000 and the first 1,000 samples were discarded as burn-in. Convergence of the chain was tested by comparing results for two chain lengths on a subset of the scenarios. In order to improve MCMC mixing, the SNP genotype matrix **Z** was centered so that **Z1** = **0**. The full conditional distributions for BayesN are given in S2 Appendix. Statistical inference for the parameters and predictions of breeding values were based on posterior means computed from the post-burn-in Markov chain. Accuracy of prediction was calculated as the correlation between genomic estimated breeding values (GEBV) and true breeding values (TBV) for validation individuals. Paired t-tests were used to compare mean accuracies between marker panels or between methods, where pairs were based on the two accuracies of prediction obtained for the same replicate.

### Data simulations

**Fig 1:**
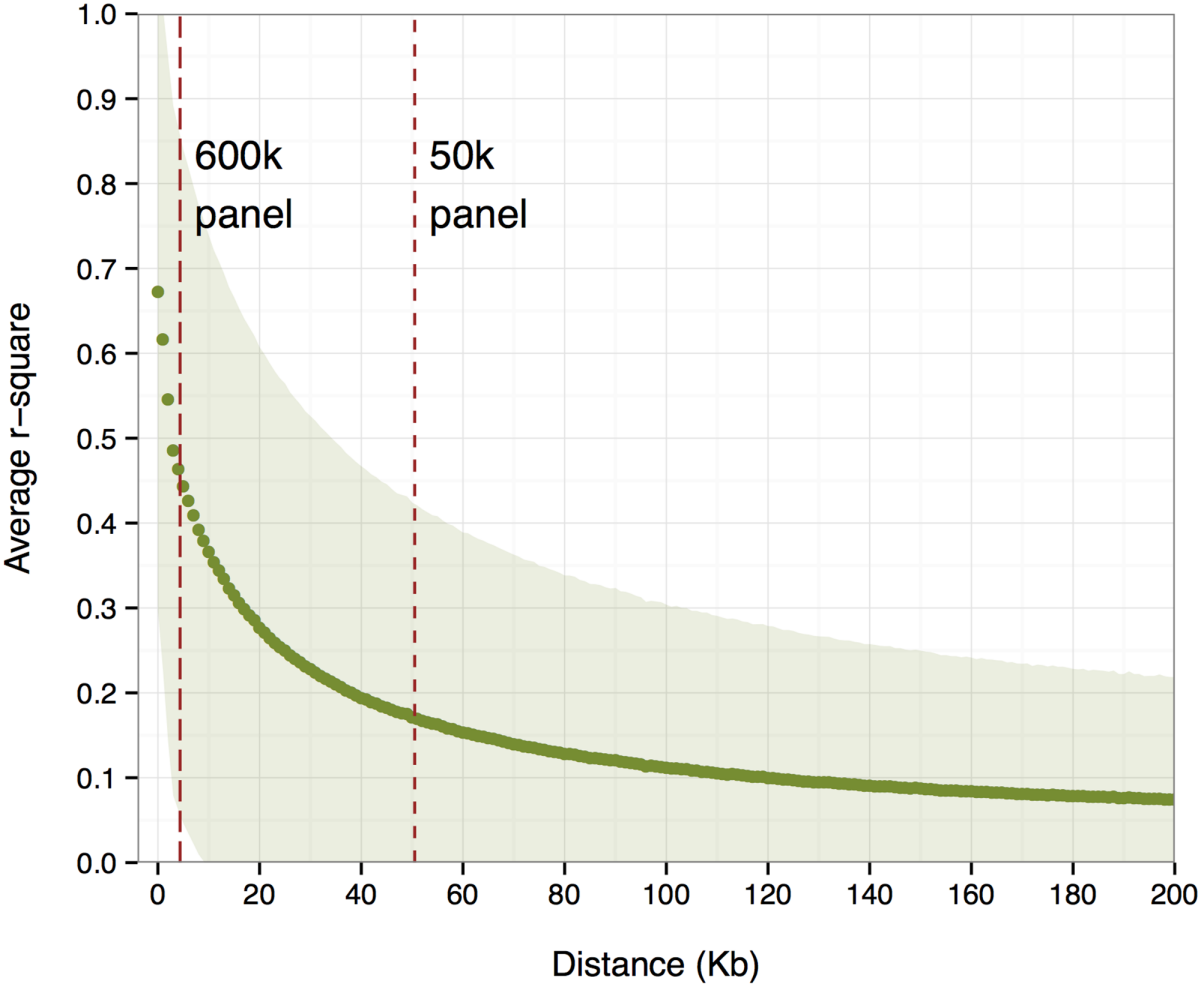
Average linkage disequilibrium (LD) between any two SNPs within 0.2 Mb distance across the genome. The shaded areas indicate one standard deviation departures from the average. The average distance between adjacent SNPs for the MD 50k and HD 600k SNP panel are indicated as broken vertical lines.

Our simulations were based on 948 Aberdeen Angus beef cattle that were genotyped with the Illumina 777K BovineHD BeadChip [21]. These records were obtained from an existing industry database, so no specific animal care and use committee approvals were required. The HD chip included 774,268 SNPs on 30 chromosomes, among which 609,870 segregating SNPs with MAF > 0.05 were used in the analyses referred to as comprising a 600k HD panel. A 50k MD panel containing 51,094 SNPs was obtained by selecting every 12th SNP from the 600k HD panel based on the physical map position for each SNP provided by Illumina. The average distance between adjacent SNPs was 4.4 kilo-base (kb) for the 600k HD panel and 50.5 kb for the 50k MD panel. The whole genome was divided into 2,649 non-overlapping 1 Mb windows, with about 20 SNPs per window for the 50k panel and 230 SNPs per window for the 600k panel, or 12,813 non-overlapping 0.2 Mb windows, with about 5 SNPs per window for the 50k panel and 50 SNPs per window for the 600k panel. The haplotype phase for each genotype was resolved via FImpute [22] without accounting for pedigree information. Fig 1 shows the average LD calculated from the haplotypes within 0.2 Mb distance, which are similar to what have been found in other cattle populations [23]. The average LD between adjacent SNPs was 0.241 in the 50k panel and 0.567 in the 600k panel. The above information was summarized in Table 1.

**Table 1:** Information for the 50k MD and 600k HD panels in the analyses.

	50k MD Panel	600k HD Panel
Number of SNPs	51,094	609,870
Distance between adjacent SNPs	50.5 kb	4.4 kb
LD between adjacent SNPs	0.241	0.567
Number of 1 Mb windows	2,649	
Number of SNPs per 1 Mb windows	19.8	229.7
Number of 0.2 Mb windows	12,813	
Number of SNPs per 0.2 Mb windows	4.6	47.5

Phased haplotypes were used to simulate SNP genotypes for 5,000 offspring by simulated random mating and allele dropping from the parents to the offspring; 4,000 of these were used for training and the remaining 1,000 were used for validation. The number of crossovers per meiosis was modeled by a binomial map function with an expectation of one crossover per Morgan [24]. Mutation was ignored. Three hundred SNPs were randomly selected according to MAF > 0.05 or < 0.05 to represent common or rare QTL, and these loci were masked from the marker panels. The QTL effects were sampled from a standard normal distribution and then divided by the realized additive genetic variance in the 5,000 offspring. The true breeding values (TBV) were the sum of the QTL genotypes ∈ {0, 1, 2} multiplied by the standardized QTL effects. Trait phenotypes with heritability 0.5 were simulated by adding random standard normal deviates to TBV. The simulation was carried out for eight replicates for each scenario of the common versus rare QTL alleles.

Our simulation scheme resulted in some training and validation animals being half sibs. Usually, ancestors and descendants are simulated for training and validation, respectively. However, repetition of such a multi-generational simulation would have required greater computational resources in our setting. We didn’t simulate a generation or time effect, therefore whether the training and validation animals are siblings or ancestors and descendants should have a negligible impact on the results of model comparison. We have verified that with simulations using only one chromosome.

## Results

The change in accuracy of prediction was negligible for 100,000 compared to 20,000 post-burn-in iterations of samples from the Markov chain. Accordingly, all subsequent results reflect the 20,000 post-burn-in samples.

### Accuracy of prediction

**Fig 2:**
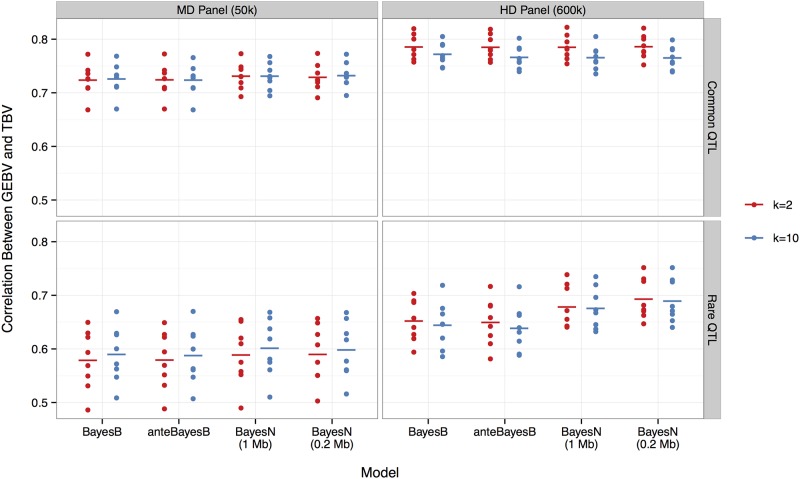
The accuracy of prediction using BayesB, anteBayesB or BayesN with 1 or 0.2 Mb windows for two values of *π* or *π*_*i*_ corresponding to 300 QTL each being associated with either 2 (red) or 10 (blue) SNP markers. Results are separated for common (row 1) versus rare (row 2) QTL alleles with the MD 50k (column 1) versus HD 600k (column 2) SNP panel. Dots represent accuracies from each of the eight replicates, and the bar indicates the mean.

Fig 2 shows the accuracy of prediction for common versus rare MAF for QTL alleles, SNP densities (50k versus 600k), assumptions for the number of SNPs (2 versus 10) associated with each QTL in the analysis, and different methods (BayesB, anteBayesB and BayesN with 1 or 0.2 Mb windows).

The accuracies were much lower when QTL alleles were all rare rather than common. The average accuracy over all methods for the 50k panel was 0.589 for the rare QTL scenario versus 0.727 for the common QTL scenario. The 600k SNP panel resulted in an increase in the average accuracy by 0.076 (12.9%, p-value < 2e-16) for the rare QTL scenario and by 0.049 (6.7%, p-value < 2e-16) for the common QTL scenario. Fitting 10 rather than 2 SNPs per QTL with the 50k panel required a lower *π* value but tended to give higher average accuracy (by 0.006 or 0.8%, p-value = 7e-7). In contrast, with the 600k panel, results were most accurate when fitting only 2 SNPs per QTL with a gain of 0.012 (1.7%, p-value = 1e-14) in average accuracy. We therefore recommend to use *k* = 2 for HD panels and *k* = 10 for MD panels in practice.

Different window sizes for BayesN gave similar results, except in the case of rare QTL alleles with the 600k panel. A small advantage was observed for BayesN over BayesB with the 50k panel. For the common QTL scenario, using BayesN with 1 Mb windows increased accuracy by 0.007 (1.0%, p-value = 0.031) for the high *π* value and by 0.005 (0.7%, p-value = 0.187) for the low *π* value. For the rare QTL scenario, using BayesN with 1 Mb windows increased accuracy by 0.010 (1.8%, p-value = 0.025) for the high *π* value and by 0.012 (2.0%, p-value = 0.009) for the low *π* value. Differences between models were negligible for the common QTL scenario with 600k SNPs, due to the high LD between SNPs and the QTL. The advantage of BayesN was maximized when all QTL were rare and the 600k panel was used. In this case, the accuracy from BayesN with 1 Mb windows was greater than that from BayesB by 0.026 (4.0%, p-value = 0.002) for the high *π* value and by 0.031 (4.9%, p-value = 9e-5) for the low *π* value. The advantage from BayesN was even greater with 0.2 Mb windows, with increases in accuracy by 0.041 (6.3%, p-value = 3e-4) for the high *π* value and by 0.045 (7.0%, p-value = 3e-4) for the low *π* value. The anteBayesB model was not competitive with BayesN and had almost the same accuracies as BayesB for all cases.

When Π and *π* were considered as unknown, similar results of prediction were observed because the posterior distribution of Π or *π* closely resembled the starting value due to a poor mixing. However, the advantage of BayesN over BayesB in prediction still holds regardless of estimation error in Π and *π*.

### Bias of prediction

**Fig 3:**
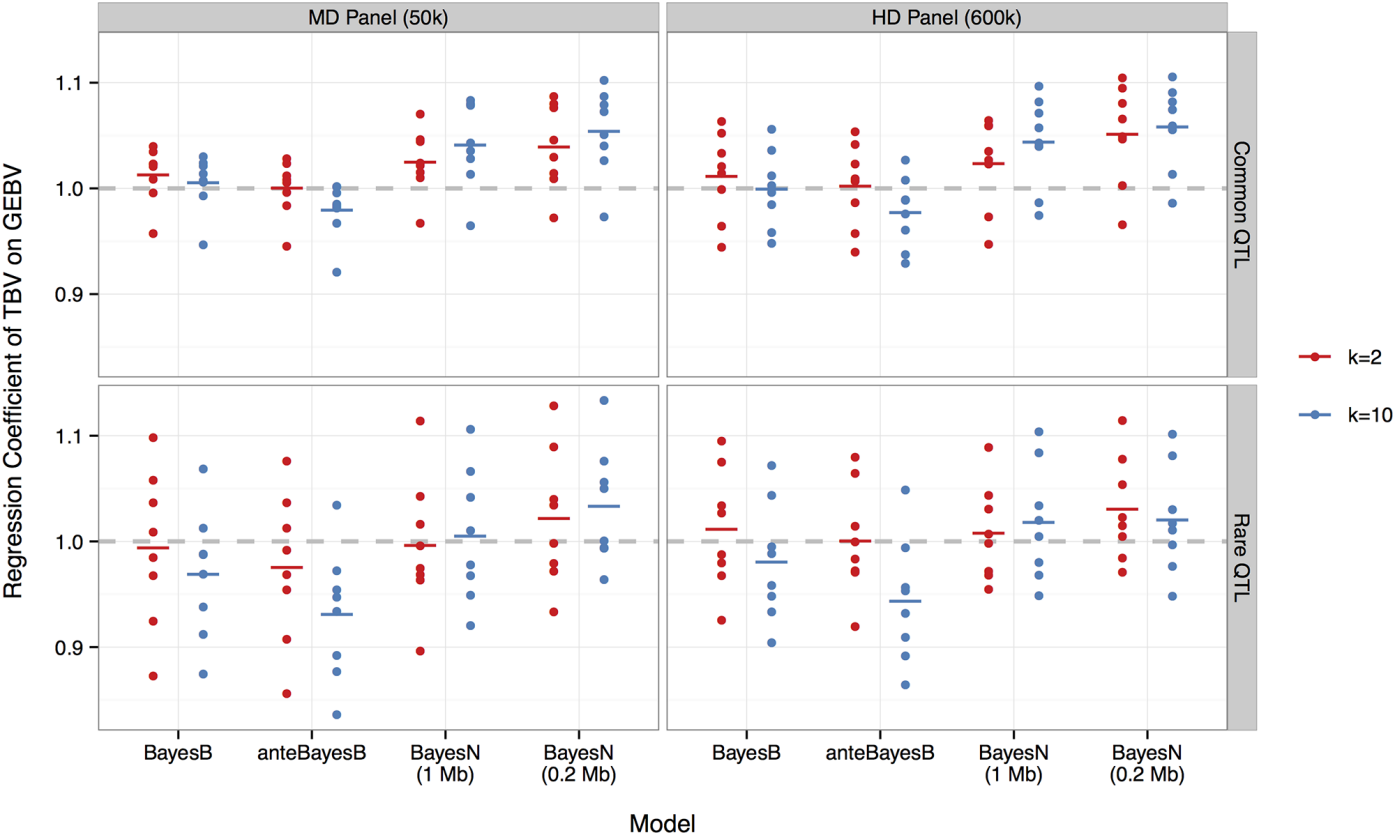
Regression coefficient of true on estimated breeding values using BayesB, anteBayesB or BayesN with 1.0 or 0.2 Mb windows for two values of *π* or *π*_*i*_ corresponding to 300 QTL each being associated with either 2 (red) or 10 (blue) SNP markers. Results are separated for common (row 1) versus rare (row 2) QTL alleles with the MD 50k (column 1) versus HD 600k (column 2) SNP panel. Dots represent regression coefficients from each of the eight replicates, and the bar indicates the mean. Regression coefficients closer to one (dashed horizontal line) reflect less prediction bias.

Bias of predictions was represented by the deviation of the regression coefficient of TBV on GEBV from one. In most cases, bias was small, in the range of −0.1 to 0.1, and not significant at p < 0.001 (Fig 3). There was more variation in estimates of bias for the rare QTL scenario (row 2) compared to the common QTL scenario (row 1). Overall, fitting 2 rather than 10 SNPs per QTL in the model resulted in lower bias, especially for anteBayesB. In the case of common QTL alleles, the GEBV from BayesN tended to be biased downward, with the mean of regression coefficients (1.024) exceeding one (p-value = 0.017) when fitting 2 SNPs per window in the model.

### Number of windows included in the model

**Fig 4:**
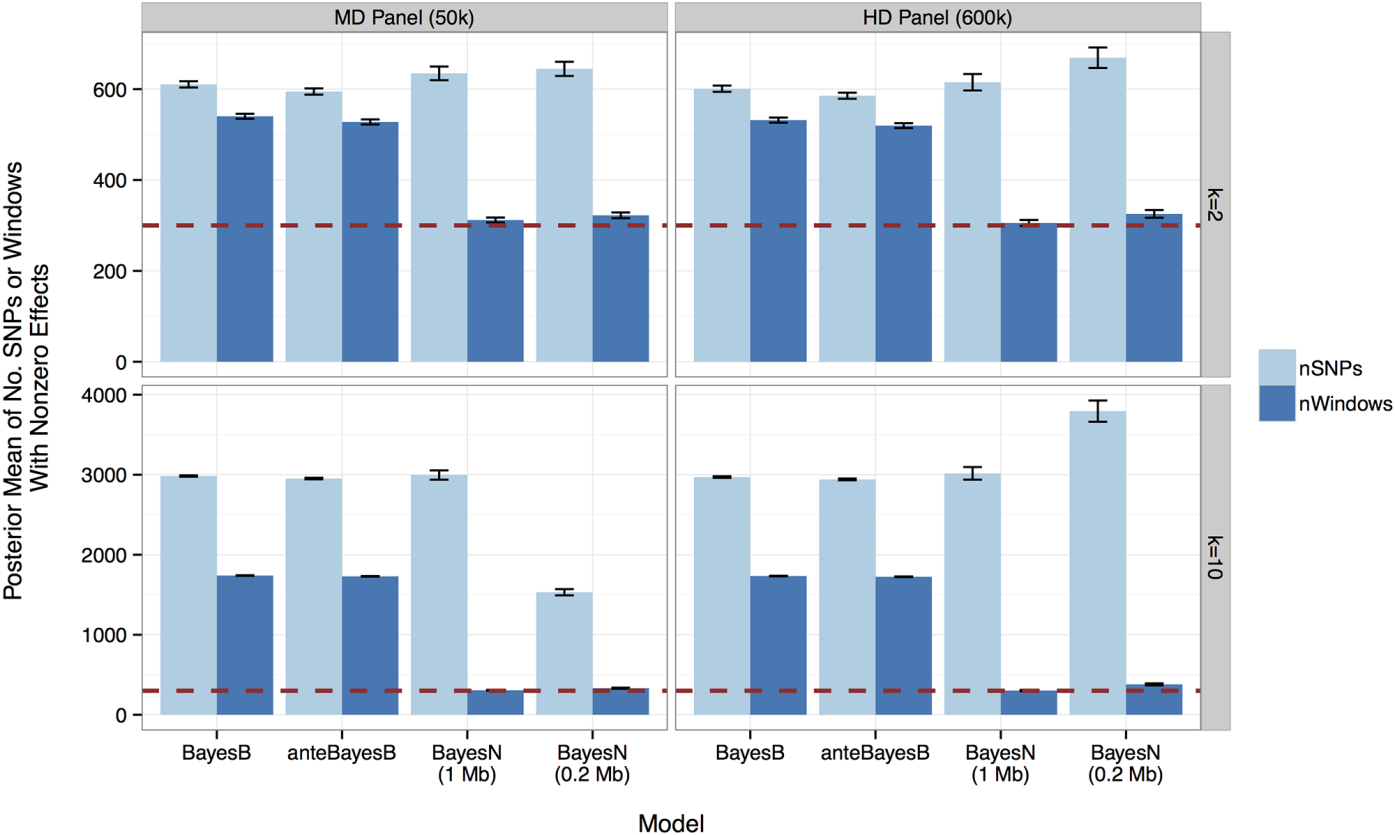
The posterior mean of number of the SNPs (light bar) and windows (dark bar) with nonzero effects from BayesB, anteBayesB or BayesN with 1.0 or 0.2 Mb windows. Results are separated for *k* = 2 (row 1) versus *k* = 10 (row 2) SNPs associated with each of the 300 QTL with the MD 50k (column 1) versus HD 600k (column 2) SNP panel. The capped error bar indicates the standard deviation of the posterior means from 8 replicates of the scenario with common and 8 replicates of the scenario with rare QTL alleles. The red dashed line shows the number of QTL simulated, which was 300.

If each window contains at most one QTL, the posterior mean of the number of windows with nonzero effects reflects the number of the detected QTL. In each MCMC sample, a window has a nonzero effect if Δ_*i*_ = 1 for BayesN, or if any SNP in that window has a nonzero effect for BayesB and anteBayesB. When Π was determined based on knowing the actual number of QTL, the posterior mean for the number of windows with nonzero effects from BayesN was always about 300, which was the number of simulated QTL, regardless of the SNP density and the number of SNPs in the model (Fig 4). In contrast, for BayesB and anteBayesB, the number of windows with nonzero effects was proportional to the number of SNPs in the model. For example, the posterior mean of the number of windows with nonzero effects for BayesB was about twice the number of QTL with the 50k panel. For BayesB with the 600k panel, the number of windows with nonzero effects was about six times the number of QTL simulated. In BayesN, each window could not fit more SNPs than existed in the window, which explains why the number of SNPs with nonzero effects was much lower for the 50k panel when 0.2 Mb windows were used, as only 4 SNPs existed per window.

### Computing time

**Fig 5:**
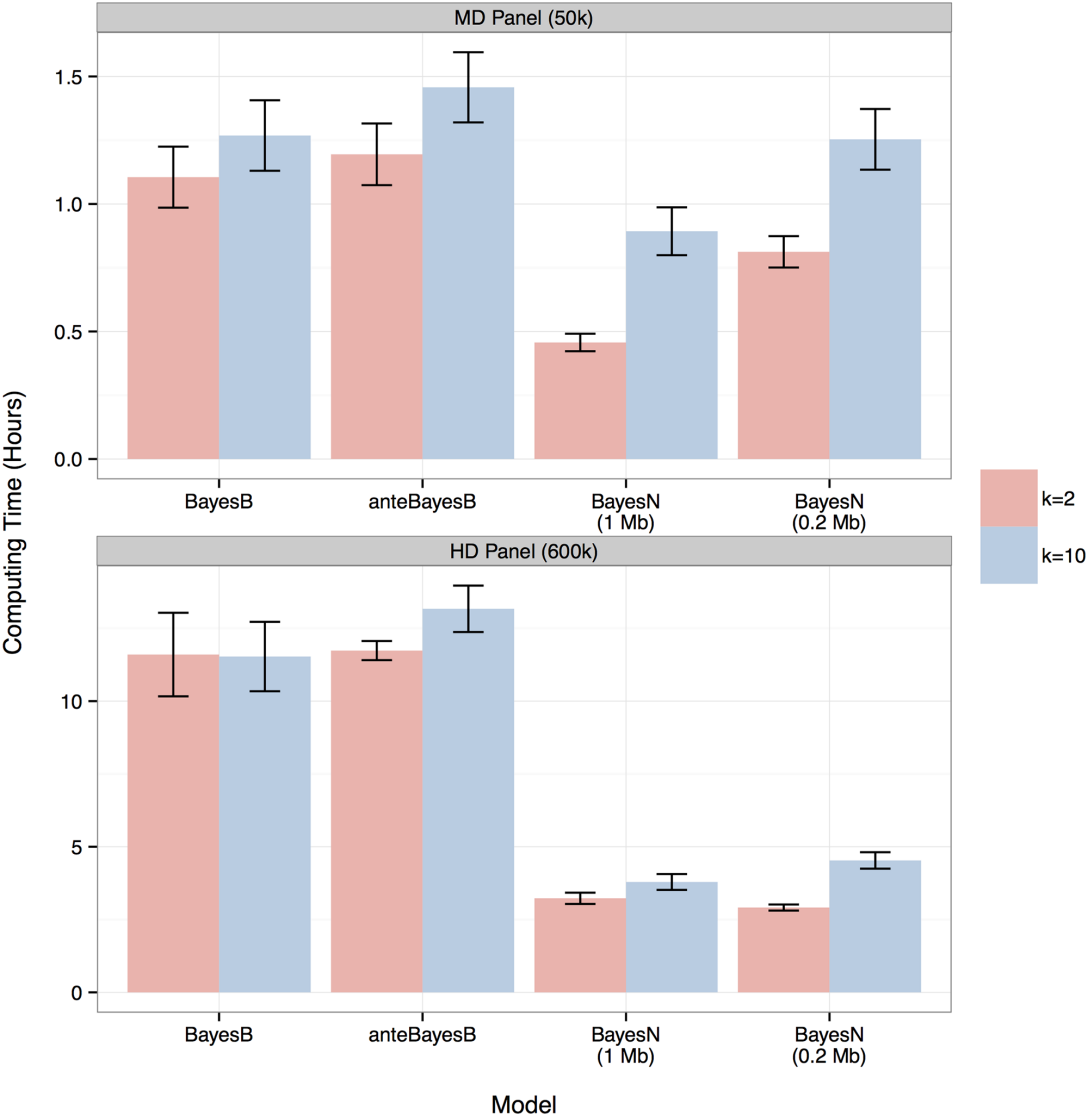
Average computing time in hours for BayesB, anteBayesB or BayesN with 1.0 or 0.2 Mb windows for two values of *π* or *π*_*i*_ corresponding to 300 QTL each being associated with either 2 (red) or 10 (blue) SNP markers. The capped error bar indicates the standard deviation from 8 replicates of common and 8 replicates of rare QTL scenarios.

Fig 5 shows the average computing time for different models implemented in C++ language on the CyEnce cluster of Iowa State University with 2.0 GHz 8-Core Intel E5 2650 processors. In general, fitting 10 SNPs (low *π* value) per QTL rather than 2 (high *π* value) demanded more computing time for any model. For BayesN, smaller windows or more SNPs per window increased computing time but this difference became marginal with high density SNPs. Using 50k SNPs, 1 Mb windows and *k* = 2, it took only 0.46 hr for BayesN, which was half the time needed for BayesB (1.1 hr, 60% reduction) or for anteBayesB (1.2 hr, 62% reduction). BayesN was even more efficient with 600k SNPs. It took only 2.9 hr for BayesN with 0.2 Mb windows, which was about one fourth of the time taken by BayesB (11.6 hr, 75% reduction) or anteBayesB (11.7 hr, 75% reduction) for *k* = 2.

### Posterior distribution of Π

**Fig 6:**
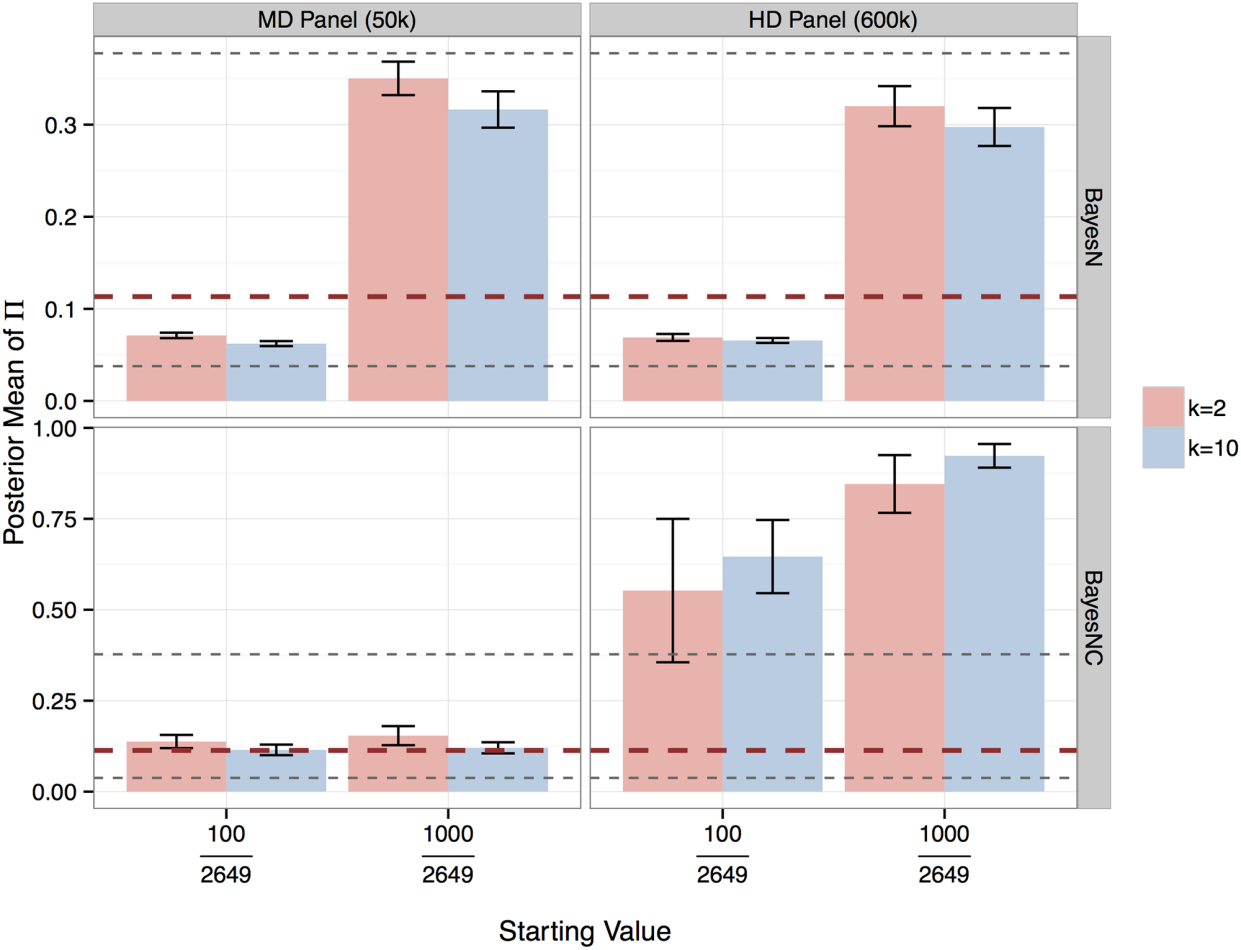
Posterior mean of Π when Π was considered as unknown in the nested model with window-specific variance $\sigma_{i}^{2}$ (BayesN) or with a common variance $\sigma_{\alpha }^{2}$ for all SNPs in the genome (BayesNC). The capped error bar indicates the standard deviation from 8 replicates of simulation for the scenario of common QTL alleles. The true Π value, *i*.*e*. the proportion of windows that contained QTL (300/2649), is shown by red dashed line, assuming at most one QTL per window. The two starting values 100/2649 and 1000/2649 are shown by black dashed lines.

If the posterior distribution of Π has a sharp peak at the true value, then considering Π as unknown would be as good as using the true value for Π in the analysis. The posterior distribution of Π was examined by computing the average value of the posterior mean and its standard deviation across replicates (Fig 6), when different starting values were used for the sampler. These starting values were also used to calculate the scale factor of variance of SNP effects, similar to the calculation in BayesB (Eq 3), but our experience is that small differences in the scale factor only have negligible influence on the results. Assuming window-specific variances $\sigma_{i}^{2}$ for the SNP effects, the posterior mean of Π largely depended on the starting value for Π for both MD and HD panels (top row). When the alternative prior (BayesNC) was used with 50k SNPs (bottom-left), where a common variance $\sigma_{\alpha }^{2}$ was assumed for all SNPs in the genome, rather than window-specific variances, the posterior distribution closely reflected the true value regardless of the starting values. In this case, fitting ten rather than two SNPs per window resulted in the posterior mean to be even closer to the true value. When 600k SNPs were used, however, BayesNC tended to fit almost all windows in the model (bottom-right).

## Discussion

### Advantages of BayesN in genomic prediction

The nested mixture model, BayesN, accounts for the dependence of SNP effects. It comprises two levels of variable selection—window selection and selection of each locus within the selected windows. Our window selection approach that collectively considers the effects of all SNPs within that window has two favorable effects. On one hand, it reduces the probability of spurious effects entering the model. On the other hand, it increases the probability for true effects entering the model, particularly in the case of high density SNPs. Compared to BayesB, noise is shrunk more heavily towards zero, while real signals are shrunk less. The second level of variable selection within a window that has been selected has two favorable effects. On one hand, it eliminates the noise that comes along with the signal due to linkage. On the other hand, fitting the selected SNPs jointly in the model expands the column space of SNP genotypes to better allow them to capture the genotypes of the QTL in the window. Although the QTL may not be in high LD with any single SNP, such as in the case rare QTL and common MAF SNPs, it may be in high LD with a linear combination of the SNPs in the window. Thus, the signal detected during window selection is refined during the subsequent within-window selection.

This may explain why in the case of common QTL alleles, BayesN had no advantage over BayesB with 600k SNPs but a small advantage with 50k SNPs (Fig 2). With 600k SNPs, some SNPs are in very high single locus LD with the QTL and these can be detected with any of the methods and, thus, additional SNPs were not needed in the model. Decreasing *π* to fit more SNPs actually impaired accuracy of prediction because this forced spurious SNPs into the model (Fig 2, top-right graph). However, with 50k SNPs, multiple SNPs from the window were needed to jointly capture the QTL effect. In contrast, in the case of rare QTL alleles, no such SNPs in high single locus LD with QTL exist, in which case BayesN was much more beneficial. This is particularly the case with 50k SNPs because multi-locus LD with rare QTL was too low to be useful, such that fitting ten SNPs per window in BayesN was slightly better than that fitting only two (Fig 2, bottom-left graph). In contrast, with 600k SNPs, there were many candidate SNPs available in a window to select from and a small set can have sufficient multi-locus LD to capture the effect of rare QTL. Thus, BayesN was specially useful with the high-density SNP panel for capturing rare QTL alleles. In other cases, BayesN was no worse than BayesB. Note that there has been controversial about whether rare or common QTL contribute most of the genetic variance with supporting evidence on both sides [7, 25].

In BayesB, SNPs that entered the model were sparsely distributed across many windows, including those containing no QTL (Fig 4). The SNPs that enter the model from outside of the QTL windows are likely to be spurious, and when spurious SNPs are included in the model, those with true effects are likely left out.

### Comparison with anteBayesB

Both BayesN and anteBayesB attempt to account for the dependence between SNP effects. However, unlike BayesN, anteBayesB did not give a higher accuracy than BayesB. In anteBayesB, the covariance between SNP effects, conditional on *t*_*j*_ in Eq (8) with a *t* prior distribution, is assumed to be specific to each marker pair. This is analogous to the so-called locus-specific variance in BayesB, conditional on which each SNP has a normal distribution. Thus, in BayesB, the marginal distribution of each SNP effect is an infinite mixture of normals. Similarly, in anteBayesB, the marginal distribution of a pair of SNP effects is an infinite mixture of bivariate normals.

In contrast to BayesB, where a SNP effect has an infinite mixture of normals, in ridge-regression BLUP, which is simply referred to BLUP in [9], a SNP effect has a univariate normal distribution. Although BayesB is expected to be perform better than BLUP as demonstrated by simulation in [9], it has been shown that the advantage of BayesB over GBLUP, which is equivalent to ridge-regression BLUP, is not observed when the training dataset is not sufficiently large [26]. As anteBayesB is even more complex than BayesB, it is not expected to be outperform BayesB when the training dataset is not sufficiently large.

In the study of Yang and Tempelman [13], where a good gain in accuracy of prediction was reported using an antedependence model, either a small number of QTL (30 QTL) was simulated such that each had a biggish effect, or the number of observations for training was comparable to or even greater than the number of markers. Jiang *et al*. [27] followed Yang and Tempelman [13]’s simulation scheme, with training size remained small (i.e. 500) but doubled number of markers (∼4k), and found that for a trait with heritability of 0.5 difference between BayesA and anteBayesA in prediction accuracy became negligible when the number of QTL increased from 30 to 300 or when LD between loci was lowered (both cases reduced QTL effects to be captured by markers). Our results on anteBayesB, with a practical problem dimension, are in agreement with those from an analysis of 30k SNPs on ∼4k pigs [28], where the antedependence model was about equal to GBLUP but inferior to BayesA and BayesB for accuracy of prediction. Another possible reason for the poor performance of anteBayesB in this study could be inadequacy of capturing multiple nonadjacent SNPs in LD with the QTL, as the covariance decayed fast with the recursive decay function. On the contrary, BayesN better captured wide-range LD within a window with a parsimonious parameterization.

### Comparison with a haplotype model

It has been reported that fitting haplotypes improves the accuracy of genomic prediction [29, 30], especially for rare QTL [31]. Using simulated data comprising sufficiently high density SNPs, QTL alleles were found to be in complete concordance with haplotype alleles [31]. BayesN and a model that fits haplotype alleles both have the same goal of reducing the number of parameters and exploit multi-locus LD. However, knowledge of haplotype phase is not needed for BayesN. In addition, the haplotype model fits the whole window, which may result in more haplotype alleles than SNPs. Different haplotype alleles may be associated with the same QTL allele, which unnecessarily increases the number of effects to be fitted. Rare haplotype alleles may also be due to genotyping and imputation errors. In contrast, with BayesN, only those few SNPs with signals are selected and fitted jointly, which may reduce the model complexity as compared with the haplotype model. Empirical comparisons between BayesN and the haplotype model will be investigated in a following study.

### Application with mixed SNP density

Saatchi and Garrick [32] showed that using a mixed density SNP panel, where genotypes were imputed only in pre-identified candidate regions, gave higher accuracies of prediction than using the original panel without imputation. In that particular beef cattle dataset, 50k genotypes were enriched with imputed 770k SNPs only in windows contributing the highest proportion of genetic variance in a genome-wise association study. The construction of a mixed density panel takes two steps. First, whole genome scans are carried out using an MD panel to identify regions that explain a significant proportion of genetic variance. Next, the identified regions are enriched with HD SNPs or sequence variants. For such mixed density analyses, BayesN has unique advantages. With BayesB, the probability of a SNP to have a nonzero effect, *π*, is common to all SNPs, which is subject to the SNP density across the entire genome. In the case of mixed density, *π* will increase if more regions are enriched with high density SNPs, and vice versa. With BayesN, due to the window-specific probability of inclusion *π*_*i*_, the effect of a change in “local” density is constrained within the window. In other words, signals from the identified regions will be independently enhanced with enriched SNP density. Furthermore, BayesN can be easily modified to fit different numbers of SNPs per window for the low and high density regions, as it has been observed that fewer SNPs were needed to capture the QTL effects with high density than low density SNPs (Fig 2).

### Computational benefit

Bayesian methods for genomic prediction commonly rely on MCMC sampling for statistical inference. The computing time for these methods increases linearly with the number of SNPs and the number of observations. Non-MCMC algorithms, such as EM algorithm [33–35], variational Bayesian approximation [36], or other iterative methods [37, 38]. have been developed to speed up the analysis of large datasets. But the accuracy of prediction was either lower than or close to BayesB or the MCMC-based counterpart. Another strategy to reduce computing time is to use parallel computing. Cheng *et al*. [39] parallelized MCMC using independent Metropolis-Hastings samplings. Fernando *et al*. [40] distributed the computation for Gibbs sampling across cores and nodes. Significant speedup was achieved when genotypes were available for millions of individuals, which could occur with real or imputed genotypes. The nested mixture model is another strategy to reduce computing time by reducing the effort to sample if SNPs have zero effect. In BayesB, the conditional probability of zero effect is computed for every SNP, whereas in BayesN, all SNPs in a window have zero effect if the window has zero effect, which depends only on a subset of SNPs with *δ*_*j*_ = 1 (Eq 6). The reduction in computing time was proportional to the SNP density (Fig 5); compared with BayesB, computing time was decreased two fold with 50k SNPs and four fold with 600k SNPs. Thus, the computational efficiency of BayesN over BayesB will even be greater for sequence variant analyses.

### Alternative prior for BayesN

Most of the results in this study were based on analyses where *π* or Π were assumed to be known. It is difficult to infer *π* when BayesB-like priors are used for marker effects [12], because BayesB has two methods to shrink the effect of a SNP; either by fitting the effect with a small variance or by including the effect in the zero effect distribution [12, 41]. The fraction of SNPs with zero effects, *π*, can be considered as unknown in method BayesC and estimated from the data because all fitted markers share the same variance ratio [11]. BayesN with BayesB-like priors between windows, i.e. window-specific variances, has a similar problem of estimating Π for windows, as the problem of estimating *π* for SNPs in BayesB. The posterior mean of Π was heavily influenced by the starting value of Π (Fig 6, top row). This lack of “Bayesian learning” can be attributed to the difficulty of sampling Δ_*i*_ due to having window-specific variances. That is, a window can have a small or zero effect by either a very small variance or a high Π value. The alternative prior, BayesNC, removed this “uncertainty” with 50k SNPs (Fig 6, bottom-left), where the effects of SNPs across windows shared a common variance such that they jointly follow a multivariate-t distribution. The reason for the overestimation of Π in BayesNC with 600k SNPs was that, with 4k observations versus 600k SNPs, the model was so overparameterized such that windows could enter the model by picking up residual effects. The same phenomenon was observed when fewer observations were used for 50k SNPs. This problem will dissipate with more data or less SNPs. One suggestion is to use the value of Π estimated from 50k SNPs and consider that value as fixed in the analysis with 600k SNPs. It is easy to see that the number of windows rather than the number of SNPs with nonzero effects is a better estimator for the number of QTL affecting the trait.

### SNP selection incorporating biological information

In our application of BayesN, all windows had equal width of 1 or 0.2 Mb. A better choice could be to define windows with unequal widths. Beissinger *et al*. [42] adopted smoothing spline techniques to define window boundaries. Alternatively, they might be determined by QTL regions, haplotype diversity, recombination hotspots or functional information of SNPs. For instance, SNPs in a intergenic region could be grouped into a single or several windows depending on the size of the intergenic region. A gene region can be partitioned into intronic and exonic windows, and the SNPs in an exon could be further partitioned into the coding or non-coding segments. Synonymous and non-synonymous SNPs could be considered to be in separate windows. MacLeod *et al*. [43] developed BayesRC to incorporate biological information on SNPs for QTL discovery. They classified SNPs prior to the analysis based on the common biological properties such as non-synonymous SNPs versus synonymous SNPs. The classified SNPs were then fitted to a model where the SNP effect is a mixture of four normals. Compared with the method without biological information, the BayesRC method resulted in more precise mapping of QTL effects and improved accuracy of genomic prediction [43]. If each window is regarded as a “class”, BayesN is similar to BayesRC in that the mixing probabilities for a SNP effect are specific to the window or the class in both models. In addition, BayesN has variable selection on windows or classes, which allows finer classifications, as described before, to better distinguish signal from noise.

## Supporting information

S1 Fig Joint distribution of the parametric values for the effects of two flanking markers X and Y of the QTL Z given some different values of the three covariances between two markers and one QTL loci. Regression coefficients for a few thousand situations where only one or other marker was informative (horizontal and vertical lines) or both markers had the same or opposite covariance with the QTL (ascending or descending diagonal lines).
(TIFF)

S2 Fig Joint distribution of the parametric values for the effects of two flanking markers X and Y of the QTL Z given numerous different values of the three covariances between two markers and one QTL loci. Regression coefficients for millions of possible covariance matrices among two markers and one QTL loci.
(TIFF)

S3 Fig Joint distribution of the parametric values for the effects of two flanking SNPs for each of the 300 QTL in one replicate of simulation, in the case of common QTL alleles. (TIFF)

S4 Fig Joint distribution of the parametric values for the effects of two flanking SNPs for each of the 300 QTL in one replicate of simulation, in the case of rare QTL alleles. (TIFF)

S1 Appendix Correlation between genotypes causes dependence of marker effects. (PDF)

S2 Appendix Markov chain Monte Carlo implementation strategy for BayesN. (PDF)
