# Supplementary figures and images for: A nested mixture model for genomic prediction using whole-genome SNP genotypes
Source: PLoS One. 2018 Mar 21;13(3):e0194683. doi: 10.1371/journal.pone.0194683 (PMC5862491; doi:10.1371/journal.pone.0194683)

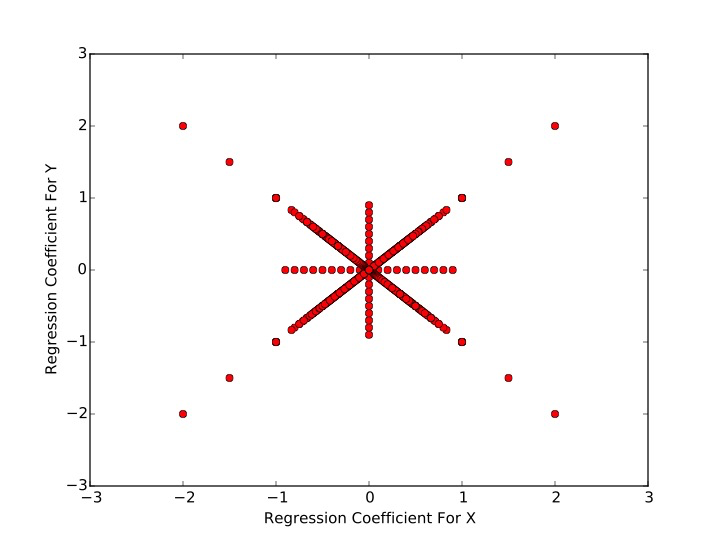

Supplement: S1 Fig — Regression coefficients for a few thousand situations where only one or other marker was informative (horizontal and vertical lines) or both markers had the same or opposite covariance with the QTL (ascending or descending diagonal lines). (TIFF) [file pone.0194683.s001.tiff]

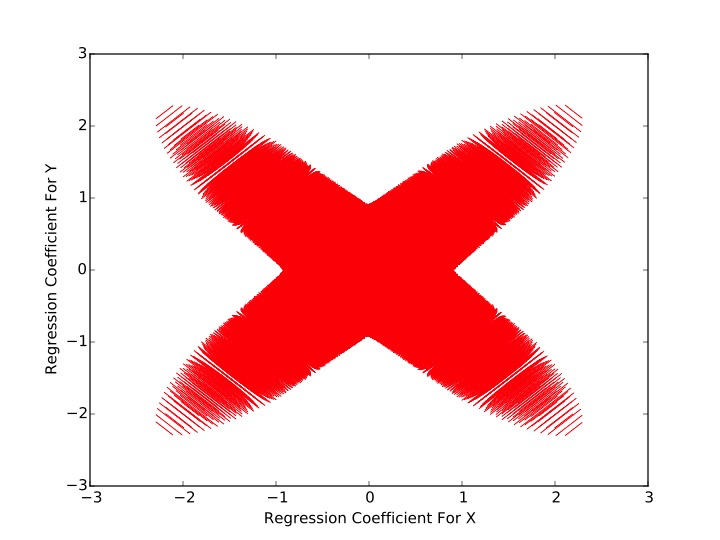

Supplement: S2 Fig — Regression coefficients for millions of possible covariance matrices among two markers and one QTL loci. (TIFF) [file pone.0194683.s002.tiff]

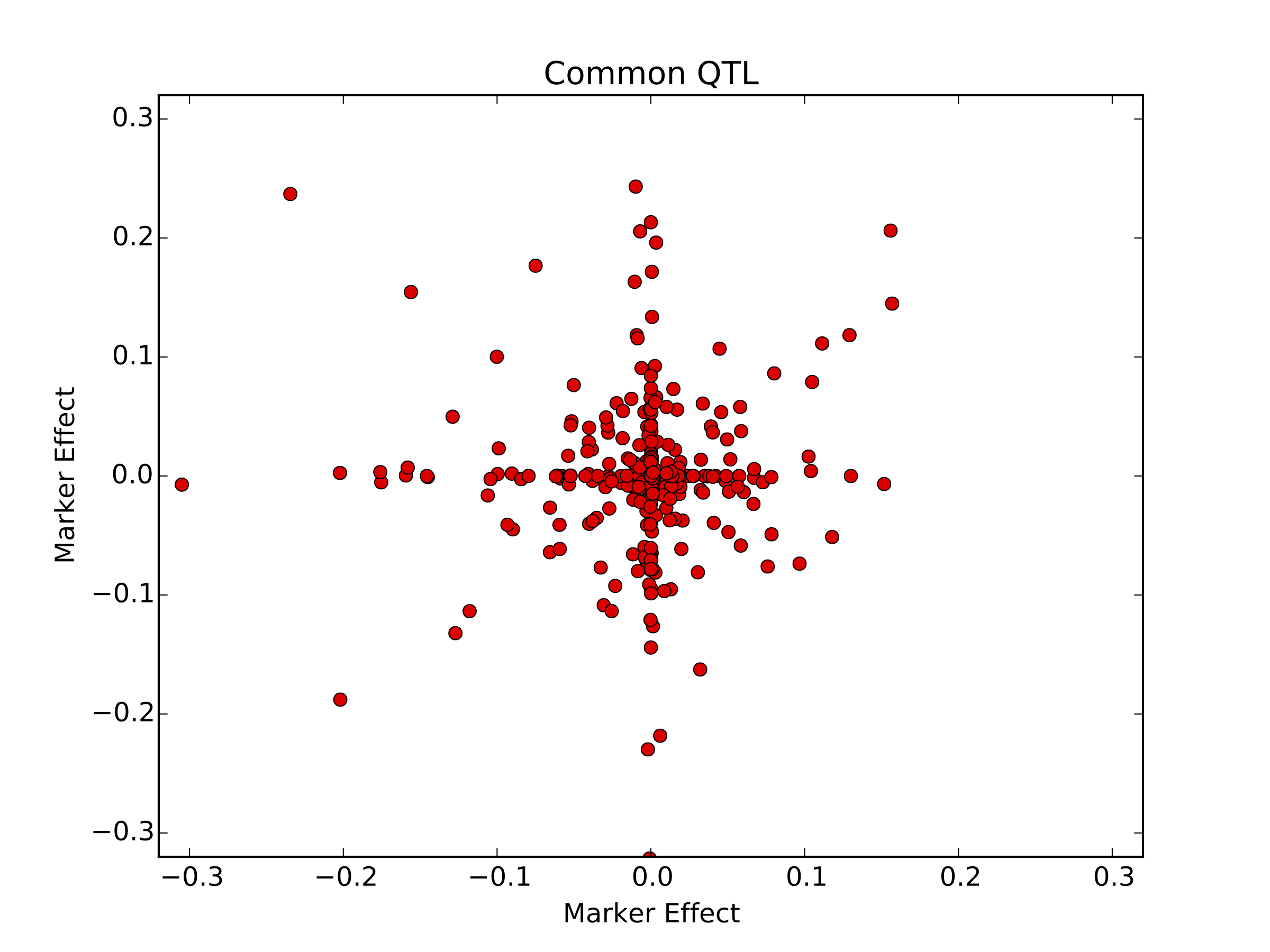

Supplement: S3 Fig — (TIFF) [file pone.0194683.s003.tiff]

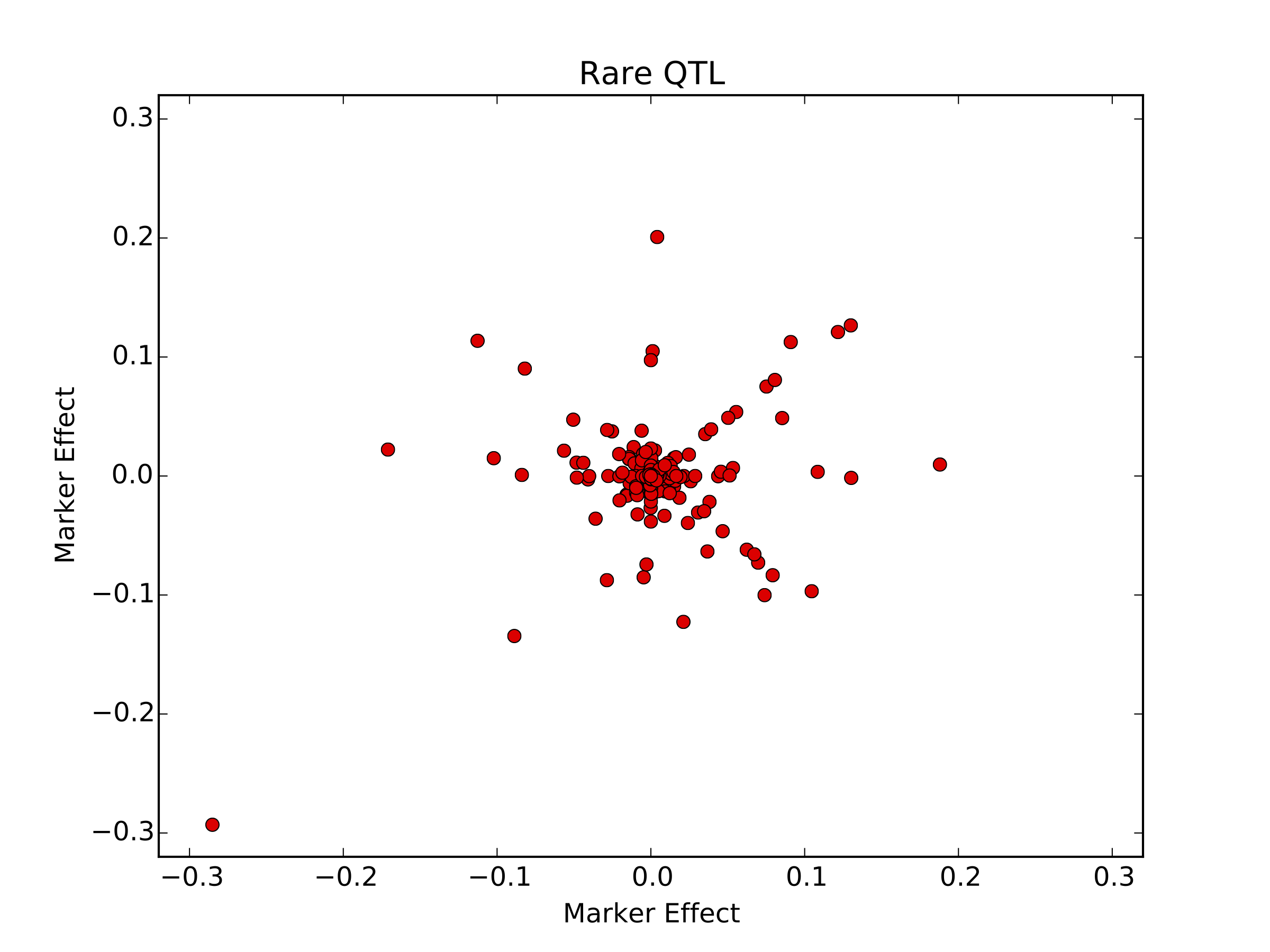

Supplement: S4 Fig — (TIFF) [file pone.0194683.s004.tiff]
