# Supplementary material for: A nested mixture model for genomic prediction using whole-genome SNP genotypes
Source: PLoS One. 2018 Mar 21;13(3):e0194683. doi: 10.1371/journal.pone.0194683 (PMC5862491; doi:10.1371/journal.pone.0194683)
Supplement: S1 Appendix — (PDF) [file pone.0194683.s005.pdf]

S1 Appendix: Correlation between genotypes causes dependence of marker effects

We show below that the correlation between marker and QTL genotypes results in dependence of marker effects. Consider a QTL and two flanking markers each with allele frequency 0.5. The cosegregation of markers and the QTL creates covariance among their genotypes. Let X and Y represent two marker genotype covariates and Z a QTL covariate. Let the covariance matrix for these three variables be

$$\mathbf{V} = \mathrm{Var}\begin{bmatrix} X \\ Y \\ Z \end{bmatrix} = \frac{1}{2}\begin{bmatrix} 1 & \sigma_{XY} & \sigma_{XZ} \\ \sigma_{XY} & 1 & \sigma_{YZ} \\ \sigma_{XZ} & \sigma_{YZ} & 1 \end{bmatrix} = \begin{bmatrix} \mathbf{P} & \mathbf{c} \\ \mathbf{c}' & 1 \end{bmatrix}. \tag{1}$$

The values on the diagonals would vary if gene frequencies departed from 0.5. Off-diagonal elements of $\mathbf{V}$ depend on LD between the loci. Let a be the QTL effect. Then, the best linear predictor of the breeding value Za given X and Y is

$$\mathrm{BLP}(Za) = \mu_g + b_X(X - \mu_X) + b_Y(Y - \mu_Y),$$

where the regression coefficients b_X and b_Y are

$$\begin{bmatrix} b_X \\ b_Y \end{bmatrix} = \mathbf{P}^{-1}\mathbf{c}a. \tag{2}$$

These regression coefficients are parametric values for the marker effects when marker-QTL genotypes are randomly sampled with a covariance matrix $\mathbf{V}$. Marker effects become random variables when their parametric values are computed across random samples of $\mathbf{V}$. Suppose $a = 1$. S1 Fig shows the parametric values for the marker effects given different covariance structures by varying some covariances from -0.9 to 0.9 with step size of 0.1. When one or other flanking markers was uninformative, *i.e.* σ_{XZ} or $\sigma_{YZ} = 0$ and $\sigma_{XY} = 0$, only the informative marker contributed to the prediction, and this resulted in a vertical (Y informative) or horizontal (X informative) line in the figure. When two markers had the same magnitude of LD with the QTL, the correlation between the marker effects was 1 or -1 corresponding to the same ($\sigma_{XZ} = \sigma_{YZ}$) or opposite ($\sigma_{XZ} = -\sigma_{YZ}$) direction of covariances. S2 Fig shows the parametric values of marker effects computed from a collection of variance-covariance matrices obtained by independently varying each of the three covariances in $\mathbf{V}$ from -0.9 to 0.9 with step size of 0.01. The collection of $\mathbf{V}$ matrices generated in this manner would include negative definite matrices which would have at least one negative eigenvalue, and these were identified and discarded because they cannot represent covariance matrices. Each pair of marker effects can be classified into a positively or negatively correlated population, although the overall correlation for all pairs was zero. Holding any two covariances constant and letting the remaining one vary resulted in the marker effects having a correlation of 1 or -1. Thus, each of the two populations was a collection of many parallel diagonal lines.

As shown above, if we only consider marker pairs that have any one of the covariances in $\mathbf{V}$ being random, *e.g.* markers having the same LD with the QTL but different LD between them, the correlation between the marker effects would be 1, -1 or not defined. If we consider marker pairs that have more than one covariance in $\mathbf{V}$ being random, *e.g.* two markers randomly sampled from the genome, the marker effects would be dependent but the correlation between them would be zero.

We calculated the covariances i.e., LD between every one of the 300 QTL and its two flanking markers in one replicate of the simulation and calculated the regression coefficients using Eq (2) with estimated $\mathbf{P}$ and $\mathbf{c}$ from the SNP genotypes. The same pattern of cross was observed for common (S3 Fig) or rare (S4 Fig) QTL alleles and shows similarity to both S1 and S2 Figs, recognizing the real data comprises only 300 realizations of the millions of covariance matrices represented in S2 Fig.
