# Supplementary material for: A nested mixture model for genomic prediction using whole-genome SNP genotypes
Source: PLoS One. 2018 Mar 21;13(3):e0194683. doi: 10.1371/journal.pone.0194683 (PMC5862491; doi:10.1371/journal.pone.0194683)
Supplement: S2 Appendix — (PDF) [file pone.0194683.s006.pdf]

S2 Appendix: Markov chain Monte Carlo implementation strategy for BayesN

The sampling scheme in BayesN is described here. Gibbs sampling is used to sample the unknowns. The joint posterior distribution of all unknown parameters in the model can be written as

$$\begin{aligned} f\left(\mu, \boldsymbol{\alpha}, \boldsymbol{\delta}, \boldsymbol{\Delta}, \boldsymbol{\sigma}^2, \sigma_e^2 \middle| \mathbf{y}\right) \propto\; & f\left(\mathbf{y} \left| \mu, \boldsymbol{\alpha}, \boldsymbol{\delta}, \boldsymbol{\Delta}, \boldsymbol{\sigma^2}, \sigma_\mathbf{e}^\mathbf{2} \right.\right) f(\mu) \\ & \prod_{i=1}^{w} \prod_{j=1}^{m_i} f\left(\alpha_{ij} \left| \sigma_i^2 \right.\right) \\ & \prod_{i=1}^{w} \prod_{j=1}^{m_i} f\left(\delta_{ij} \left| \pi_i \right.\right) \\ & \prod_{j=1}^{m_i} f\left(\Delta_i \middle| \Pi\right) \\ & \prod_{i=1}^{w} f\left(\sigma_i^2 \left| \nu_\alpha, S_\alpha^2 \right.\right) \\ & f(\sigma_e^2 \left| \nu_e, S_e^2 \right.), \end{aligned}$$

where

$$\begin{aligned} f\left(\mathbf{y} \left| \mu, \boldsymbol{\alpha}, \boldsymbol{\delta}, \boldsymbol{\Delta}, \boldsymbol{\sigma^2}, \sigma_\mathbf{e}^\mathbf{2} \right.\right) &= \mathrm{MVN}\left(\mathbf{1}\mu + \sum_{i=1}^{w} \sum_{j=1}^{m_i} \mathbf{Z}_{ij} \alpha_{ij} \delta_{ij} \Delta_i, \mathbf{I}\sigma_e^2\right), \\ f(\mu) &= \mathit{constant}, \\ f\left(\alpha_{ij} \left| \sigma_i^2, \delta_{ij}, \Delta_i \right.\right) &= \mathrm{N}\left(0, \sigma_i^2\right), \\ f\left(\delta_{ij} \left| \pi_i \right.\right) &= \pi_i^{(1-\delta_{ij})} (1-\pi_i)^{\delta_{ij}}, \\ f\left(\Delta_i \middle| \Pi\right) &= \Pi^{(1-\Delta_i)} (1-\Pi)^{\Delta_i}, \\ f\left(\sigma_i^2 \left| \nu_\alpha, S_\alpha^2 \right.\right) &= \nu_\alpha S_\alpha^2 \chi_{\nu_\alpha}^{-2}, \\ f(\sigma_e^2 \left| \nu_e, S_e^2 \right.) &= \nu_e S_e^2 \chi_{\nu_e}^{-2}. \end{aligned}$$

Sampling fixed effect μ

The fixed effect μ is sampled from the full conditional:

$$f\left(\mu \left| \mathbf{y}, ELSE \right.\right) = \mathrm{N}\left(\frac{\mathbf{1}'\left(\mathbf{y} - \mathbf{Zu}\right)}{n}, \frac{\sigma_e^2}{n}\right).$$

Sample window indicator variable Δ_i

The indicator variable Δ_i for the window in or out of the model is sampled from the full conditional probability for $\Delta_i = 1$. Suppose

$$
\begin{aligned}
\mathbf{w}_i &= \mathbf{y} - \mathbf{1}\mu - \sum_{i' \neq i} \mathbf{Z}_{i'} \mathbf{u}_{i'} \\
&= \mathbf{Z}_i \mathbf{u}_i + \mathbf{e},
\end{aligned}
$$

where $\mathbf{Z}_i$ is the sub-matrix of SNP genotypes for window i and $\mathbf{u}_i$ is the vector of the SNP effects defined in Eq. (6). Then,

$$
\begin{aligned}
\Pr\left(\Delta_i = 1 \left| \mathbf{y}, ELSE \right.\right) &= \frac{f\left(\mathbf{w}_i \left| \Delta_i = 1, \mathbf{u}_i, \sigma_e^2 \right.\right) \Pr\left(\Delta_i = 1 \right| \Pi)}{f\left(\mathbf{w}_i \left| \mathbf{u}_i, \sigma_e^2 \right.\right)} \\
&= \frac{f\left(\mathbf{w}_i \left| \Delta_i = 1, \mathbf{u}_i, \sigma_e^2 \right.\right) (1 - \Pi)}{f\left(\mathbf{w}_i \left| \mathbf{u}_i, \sigma_e^2 \right.\right)},
\end{aligned}
$$

where

$$
f\left(\mathbf{w}_i \left| \mathbf{u}_i, \sigma_e^2 \right.\right) = f\left(\mathbf{w}_i \left| \Delta_i = 0, \mathbf{u}_i, \sigma_e^2 \right.\right) \Pi + f\left(\mathbf{w}_i \left| \Delta_i = 1, \mathbf{u}_i, \sigma_e^2 \right.\right) (1 - \Pi),
$$

and

$$
\begin{aligned}
f\left(\mathbf{w}_i \left| \Delta_i = 0, ELSE \right.\right) &= \mathrm{MVN}\left(0, \mathbf{I}\sigma_{\mathbf{e}}^{\mathbf{2}}\right), \\
f\left(\mathbf{w}_i \left| \Delta_i = 1, ELSE \right.\right) &= \mathrm{MVN}\left(\sum_{j=1}^{m_i} \mathbf{Z}_{ij} \alpha_{ij} \delta_{ij} \Delta_i, \mathbf{I}\sigma_{\mathbf{e}}^{\mathbf{2}}\right).
\end{aligned}
$$

Sampling locus indicator variable δ_{ij} and SNP effect α_{ij}

The locus indicator variable δ_{ij} and the SNP effect α_{ij} are sampled jointly for a better mixing. This is done by first sampling δ_{ij} unconditional on α_{ij} and then sampling α_{ij} conditional on δ_{ij}. Suppose

$$
\begin{aligned}
\mathbf{w}_{ij} &= \mathbf{y} - \mathbf{1}\mu - \sum_{i' \neq i} \mathbf{Z}_{i'} u_{i'} - \sum_{j' \neq j} \mathbf{Z}_{ij'} \alpha_{ij'} \delta_{ij'} \Delta_i \\
&= \mathbf{Z}_{ij} \alpha_{ij} \delta_{ij} \Delta_i + \mathbf{e},
\end{aligned}
$$

The probability for $\delta_{ij} = 1$ conditional on the data and all other parameters but α_{ij} is

$$
\Pr\left(\delta_{ij} = 1 \left| \mathbf{y}, ELSE\ BUT\ \alpha_{ij} \right.\right) = \frac{f\left(\mathbf{w}_{ij} \left| \delta_{ij} = 1, \Delta_i, \sigma_i^2, \sigma_e^2 \right.\right) (1 - \pi_i)}{f\left(\mathbf{w}_{ij} \left| \Delta_i, \sigma_i^2, \sigma_e^2 \right.\right)}.
$$

Since $r_{ij} = \mathbf{Z}'_{ij} \mathbf{w}_{ij}$ is a sufficient statistic to calculate the likelihood density, the above equation can be reduced to

$$\Pr\left(\delta_{ij}=1\left|\mathbf{y},ELSE\ BUT\ \alpha_{ij}\right.\right) = \frac{f\left(r_{ij}\left|\delta_{ij}=1,\Delta_i,\sigma_i^2,\sigma_e^2\right.\right)(1-\pi_i)}{f\left(r_{ij}\left|\Delta_i,\sigma_i^2,\sigma_e^2\right.\right)}.$$

Because both α_{ij} and $\mathbf{e}$ are normally distributed, it can be shown that

$$\begin{aligned} f\left(r_{ij}\left|\delta_{ij}=1,\Delta_i,\sigma_i^2,\sigma_e^2\right.\right) &= \mathrm{N}\left(0,\left(\mathbf{Z}'_{ij}\mathbf{Z}_{ij}\right)^2\sigma_i^2\Delta_i+\mathbf{Z}'_{ij}\mathbf{Z}_{ij}\sigma_e^2\right), \\ f\left(r_{ij}\left|\delta_{ij}=1,\Delta_i,\sigma_i^2,\sigma_e^2\right.\right) &= \mathrm{N}\left(0,\mathbf{Z}'_{ij}\mathbf{Z}_{ij}\sigma_e^2\right). \end{aligned}$$

The SNP effect α_{ij} is then sampled from the full conditional:

$$f\left(\alpha_{ij}\left|\mathbf{y},ELSE\right.\right)=\mathrm{N}\left(\frac{\mathbf{Z}'_{ij}\mathbf{w}}{C_{ij}},\frac{\sigma_e^2}{C_{ij}}\right),$$

where

$$C_{ij}=\mathbf{Z}'_{ij}\mathbf{Z}_{ij}+\frac{\sigma_e^2}{\sigma_i^2}.$$

Sampling variances σ_i^2 and σ_e^2

The window-specific variance σ_i^2 is sampled from the full conditional:

$$f\left(\sigma_i^2\left|\nu_\alpha,S_\alpha^2\right.\right)=\tilde{\nu}_\alpha\tilde{S}_\alpha^2\chi_{\tilde{\nu}_\alpha}^{-2},$$

where

$$\tilde{\nu}_\alpha=m_i+\nu_\alpha$$

and

$$\tilde{S}_\alpha^2=\frac{\sum_{j=1}^{m_i}\alpha_{ij}^2+\nu_\alpha S_\alpha^2}{\tilde{\nu}_\alpha}.$$

Similarly, the residual variance σ_e^2 is also sampled from the full conditional:

$$f\left(\sigma_e^2\left|\nu_e,S_e^2\right.\right)=\tilde{\nu}_e\tilde{S}_e^2\chi_{\tilde{\nu}_e}^{-2},$$

where

$$\tilde{\nu}_e=n+\nu_e$$

and

$$\tilde{S}_e^2=\frac{(\mathbf{y}-\mathbf{Zu})'(\mathbf{y}-\mathbf{Zu})+\nu_e S_e^2}{\tilde{\nu}_e}.$$

Sampling Π

Since Π depends on $\mathbf{y}$ only through $\boldsymbol{\Delta}$, the full conditional for Π is,

$$f\left(\Pi|\boldsymbol{\Delta}\right) = \Pi^{\left(w-\Delta'\Delta+1\right)}\left(1-\Pi\right)^{\Delta'\Delta+1}.$$
